# Quality assurance of geometric accuracy based on an electronic portal imaging device and log data analysis for Dynamic WaveArc irradiation

**DOI:** 10.1002/acm2.12324

**Published:** 2018-04-06

**Authors:** Hideaki Hirashima, Yuki Miyabe, Mitsuhiro Nakamura, Nobutaka Mukumoto, Takashi Mizowaki, Masahiro Hiraoka

**Affiliations:** ^1^ Department of Radiation Oncology and Image‐applied therapy Graduate School of Medicine Kyoto University Kyoto Japan

**Keywords:** Dynamic WaveArc, electronic portal imaging device, log data, quality assurance

## Abstract

The purpose of this study was to develop a simple verification method for the routine quality assurance (QA) of Dynamic WaveArc (DWA) irradiation using electronic portal imaging device (EPID) images and log data analysis. First, an automatic calibration method utilizing the outermost multileaf collimator (MLC) slits was developed to correct the misalignment between the center of the EPID and the beam axis. Moreover, to verify the detection accuracy of the MLC position according to the EPID images, various positions of the MLC with intentional errors in the range 0.1–1 mm were assessed. Second, to validate the geometric accuracy during DWA irradiation, tests were designed in consideration of three indices. Test 1 evaluated the accuracy of the MLC position. Test 2 assessed dose output consistency with variable dose rate (160–400 MU/min), gantry speed (2.2–6°/s), and ring speed (0.5–2.7°/s). Test 3 validated dose output consistency with variable values of the above parameters plus MLC speed (1.6–4.2 cm/s). All tests were delivered to the EPID and compared with those obtained using a stationary radiation beam with a 0° gantry angle. Irradiation log data were recorded simultaneously. The 0.1‐mm intentional error on the MLC position could be detected by the EPID, which is smaller than the EPID pixel size. In Test 1, the MLC slit widths agreed within 0.20 mm of their exposed values. The averaged root‐mean‐square error (RMSE) of the dose outputs was less than 0.8% in Test 2 and Test 3. Using log data analysis in Test 3, the RMSE between the planned and recorded data was 0.1 mm, 0.12°, and 0.07° for the MLC position, gantry angle, and ring angle, respectively. The proposed method is useful for routine QA of the accuracy of DWA.

## 1 INTRODUCTION

Intensity‐modulated radiotherapy (IMRT) is an effective method for achieving high dose conformity for the target in radiotherapy.^1^ Volumetric‐modulated arc radiotherapy (VMAT) has been developed to reduce the treatment time by allowing additional degrees of freedom, such as variations in the gantry speed and dose rate, as well as dynamically changing the shape of the field,^2^, ^3^, ^4^, ^5^, ^6^ while noncoplanar VMAT has further improved the dose distribution.^7^, ^8^, ^9^

Dynamic WaveArc (DWA), a new function incorporated into Vero4DRT instruments (MHI‐TM2000; Mitsubishi Heavy Industries, Ltd., Hiroshima, Japan, and Brainlab AG, Feldkirchen, Germany), could be used to perform novel three‐dimensional noncoplanar irradiation.^10^, ^11^ The Vero4DRT is composed of an O‐ring gantry that is designed to rotate ±180°, and can itself also rotate ±60° around the vertical axis. The MLC design is a single‐focus type, has 30 pairs of 5 mm thick leaves at the isocenter, and produces a maximum field size of 150 × 150 mm^2^. The DWA technique is a beam delivery method designed to maximize the versatility of the Vero4DRT by synchronizing the noncoplanar movement of the gantry/ring (G/R) with the optimization of the dynamic multileaf collimator (MLC). It is a similar technique to noncoplanar VMAT, and noncoplanar beam directions are capable of being selected from a list of preinstalled trajectories in the treatment planning system. Noncoplanar VMAT produces a high conformal dose distribution, but its delivery is inefficient because it involves rotating the patient couch.^12^, ^13^, ^14^ One of the main characteristics of the DWA technique is continuous noncoplanar VMAT without the requirement to move the couch. To maximize the benefits of the DWA approach, the Vero4DRT system incorporates the following capabilities: variable dose rate, variable gantry speed, variable ring speed, and dynamic MLC movement, with the expectation that these will optimize dose conformity, delivery efficiency, accuracy, and reliability.

DWA is a complex irradiation technique, and it is important to ensure that the device is operating correctly. Several studies have reported the geometric accuracy for DWA irradiation. Sato et al. comprehensively examined machine‐limiting accuracy during DWA irradiation in a number of situations using various dose rates, G/R angle positions, and speeds.^15^ Burghelea et al. developed a novel evaluation method for measuring the accuracy of the G/R position using a cube phantom with a kilovolt x‐ray imaging subsystem.^16^ This procedure is effective for both commissioning and detailed verification. On the other hand, a simple verification method that can quickly measure and automatically analyze is required for routine quality assurance (QA).

The purpose of this study was to develop a simple verification method for the routine QA of DWA irradiation. Several studies reported that an electronic portal imaging device (EPID) and log data analysis had sufficiently verified the accuracy of the mechanical uncertainty, that is, MLC and gantry position as well as delivery error, during VMAT.^17^, ^18^, ^19^ Therefore, we investigated the application of the QA method based on the EPID and log data analysis to DWA irradiation.

## 2 MATERIALS AND METHODS

### 2.A DWA QA using the EPID and log data analysis

DWA is an extension of noncoplanar VMAT and its irradiation accuracy depends on a complex combination of various factors. With respect to the mechanical restriction on a characteristic Vero4DRT with DWA irradiation, the maximum dose rate, gantry rotational speed, ring rotational speed, and MLC speed are 400 monitor units (MU)/min, 6.0°/s, 2.5°/s, and 4.0 cm/s, respectively.

Our proposed method utilized EPID images and log data analysis. Fluence profiles were evaluated using EPID images. EPID calibration of the Vero4DRT was performed in the manner specified by the manufacturer, by acquiring a flood and a dark‐field image. The amorphous silicon EPID on the Vero4DRT has a 180 × 180 mm^2^ detection area with a matrix size of 1024 × 1024; that is, 0.18 mm/pixel at the isocenter plane. EPID images were acquired at a rate of 1.75 frames/s. EPID images were analyzed by using relative values. The performance of the machine during DWA irradiation was also analyzed using two sets of log data: the G/R control log and the MLC control log. The G/R control log captured the accumulated MU, dose rate, gantry, and ring angles. Meanwhile, the MLC control log recorded the MLC positions (defined at the isocenter). They were recorded every 50 ms with the same time stamp by using the same controller. Analysis software based on Matlab version 8.6 (MathWorks Inc., Natick, MA, USA) was developed to evaluate the machine's performance automatically. All of the following tests irradiated the EPID and recorded log data that were then analyzed by the in‐house software.

### 2.B Calibration method for misaligned EPID geometry during DWA irradiation

**Figure 1:**
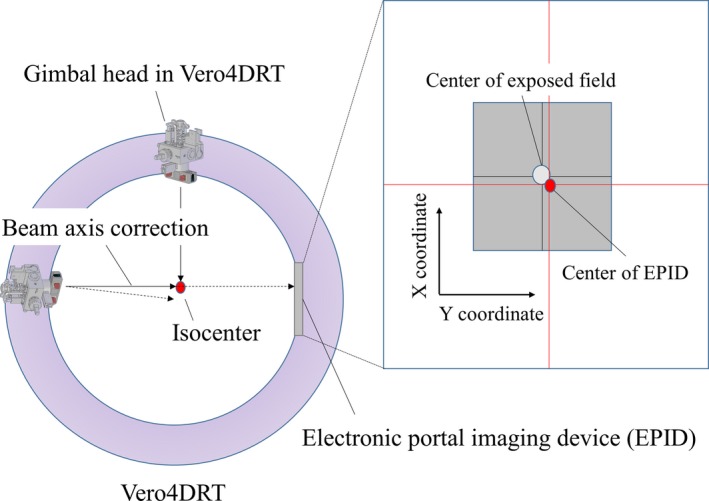
Illustration of beam axis correction through the use of a gimbal head during gantry rotation.

**Figure 2:**
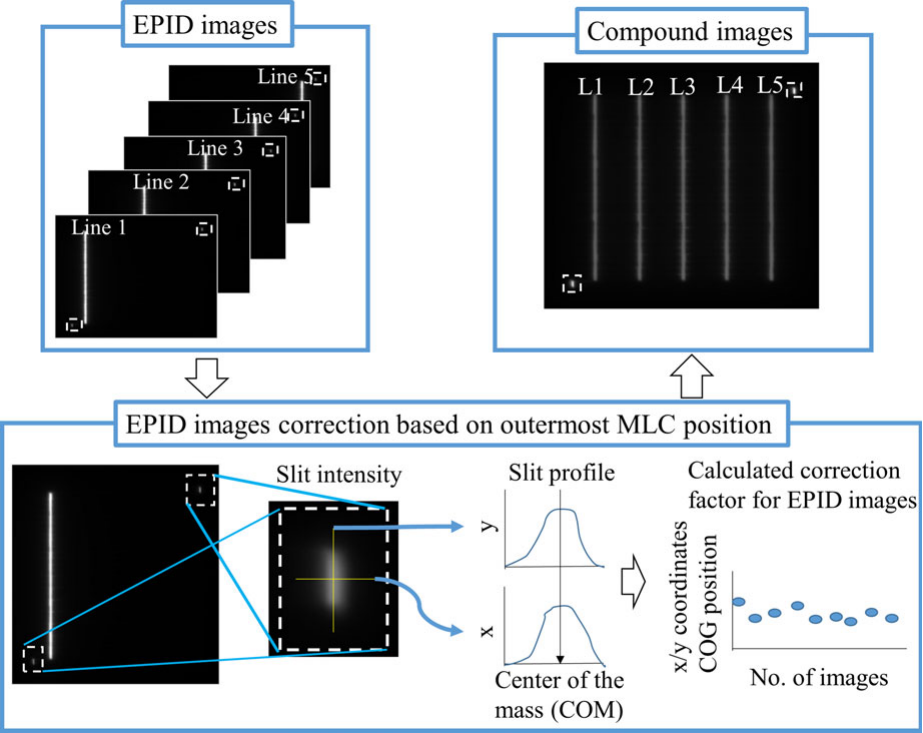
Schematic representation of the calibration method for misaligned EPID images. To correct the effect of beam axis adjustment on the MLC position in EPID images, narrow slits of the outermost MLC pair were used as a reference position on the EPID (shown in the dashed square region). Thereafter, positions of the center of the mass (COM) in the narrow slits were measured for all images. Based on the detected COM, the EPID images were translated, and a fluence map was constructed.

In general, the alignment of the megavoltage treatment beam and EPID changes during gantry rotation due to gantry and/or detector sag. In the case of the Vero4DRT, the gimbaled x‐ray head and EPID are mounted on the rigid O‐ring structure; therefore, misalignment of the beam axis is reduced. Moreover, the beam axis of each angle is sufficiently accurate owing to beam axis correction using a gimbal head^20^, ^21^, ^22^ (Fig. 1). However, to evaluate MLC positional and output accuracy using the EPID during G/R rotation, the misalignment between the center of the EPID and beam axis needs to be corrected. Therefore, an automatic calibration method for the misaligned EPID geometry was developed. The outermost MLC pairs, which are not usually used for treatment, form narrow slits and these were fixed during irradiation. The EPID images were captured in continuous mode during 360° clockwise rotation of the gantry and ±40° rotation of the ring. The O‐ring angle of DWA trajectory was limited to approximately ±40° due to gantry‐couch collision. After that, the position of the center of mass (COM) in the narrow slit was detected in each frame. According to the detected COM, the misalignment for each frame of the EPID image was then corrected. Then, all EPID images were converted to an integrated fluence map (Fig. 2).

In addition, to evaluate the detection accuracy of the EPID images, the five x‐ray slit fields in a static gantry position were irradiated by introducing an intentional error in the MLC slit width within the range 0.1–1 mm. Mean absolute error (MAE) and standard deviation (SD) of peak positions of the slit width on the exposed EPID and log data were analyzed by the in‐house software and compared to those taken without the intentional value. To eliminate the possibility of error other than the MLC position, this test was performed at static gantry.

### 2.C Development of a QA procedure for DWA irradiation

Ling et al. reported a step‐by‐step approach that examines the functional ability to deliver accurate treatments using the complex irradiation method of VMAT.^23^ In this work, the QA procedure for DWA irradiation was determined with reference to the reported method.

#### 2.C.1 Test 1: Accuracy of the MLC position

To assess the accuracy of the dynamic MLC leaf position, a picket fence test was performed at a stationary gantry angle of 0° and during DWA irradiation. The picket fence test consisted of five narrow bands with a slit width of 2 mm and spaced at intervals between two central positions of the leaf gap of 29 or 30 mm. For DWA irradiation, the gantry was rotated by 360° in a clockwise direction, while the ring was rotated by ±40°. The dose rate, gantry speed, and ring speed were 400 MU/min, 1.67, and 0.83°/s, respectively. EPID images and G/R and MLC control log data were acquired during the tests at a stationary gantry angle of 0° during DWA irradiation. The averaged central MLC profiles in the EPID images were analyzed using in‐house software (MLC number 15). The MLC profiles in the EPID images were analyzed using in‐house software. The MLC slit widths were assessed by determining the full width at half maximum (FWHM) of each of the peaks. Thereafter, displacement of slits was compared between static and DWA picket fences. The error of slit widths showed MAE and SD. At the same time, the MLC and G/R positions were then analyzed using the log data.

#### 2.C.2 Test 2: Accurate control of the dose rate, gantry speed, and ring speed

The output consistency at different dose rates, gantry speeds, and ring speeds was measured to verify the control accuracy. Four combinations of the dose rate, gantry speed, and ring speed were used to provide the same output to the four strips (see Table 1). These combinations were determined based on the following considerations: inclusion of the maximum and minimum machine limits during DWA irradiation, and commonly used conditions in clinical practice. The width of each strip was 30 mm, and the size of the interstrip gaps was 5 mm. The averaged central output profiles for DWA irradiation were compared with the open field profile at a static gantry angle of 0° (MLC number 15). DWA profiles were normalized by the maximum value of the open field. The profiles were analyzed using the in‐house software. The agreement between static and DWA irradiation was assessed based on the root‐mean‐square error (RMSE), MAE, and SD in the horizontal strip profile, except for 5 mm from the field edge. Log data were then analyzed according to the RMSE, MAE, and SD of the MLC and G/R positions. RMSE between the actual and planned values in the log data were evaluated.

**Table 1:** Irradiation parameters of the four sections in Test 2

Section no.	Dose rate (MU/min)	Gantry speed (°/s)	Ring speed (°/s)
1	160	2.4	1.2
2	200	3.0	1.5
3	333	5.0	2.5
4	400	6.0	0.6

#### 2.C.3 Test 3: Accurate control of MLC leaf speed

The output constancy was assessed as a function of the MLC leaf speed during DWA irradiation. This test used four different combinations of the dose rate, gantry speed, ring speed, and MLC speed to give the same output to the four strips (see Table 2). These combinations were also determined based on the above considerations. The width of each of the strips was 30 mm, and the size of the inter‐strip gaps was 5 mm. The DWA profiles were normalized by the maximum value of the stationary gantry position. The averaged central output profile agreement between the stationary gantry angle of 0° and DWA irradiation was also inspected using in‐house software and evaluated according to the RMSE, MAE, and SD in the exposed field, except for 5 mm from the field edge (MLC number 15). The stationary gantry angle in test 3 referred to same field using dynamic MLCs. The RMSE of the MLC and G/R motion was evaluated by log data analysis.

**Table 2:** Irradiation parameters of the four sections in Test 3

Section no.	Dose rate (MU/min)	Gantry speed (°/s)	Ring speed (°/s)	MLC speed (cm/s)
1	160	5.3	2.7	1.6
2	200	3.3	2.0	2.1
3	333	2.2	2.2	3.5
4	400	5.3	1.3	4.0

## 3 RESULTS

### 3.A EPID detection accuracy and influence of beam axis correction

**Figure 3:**
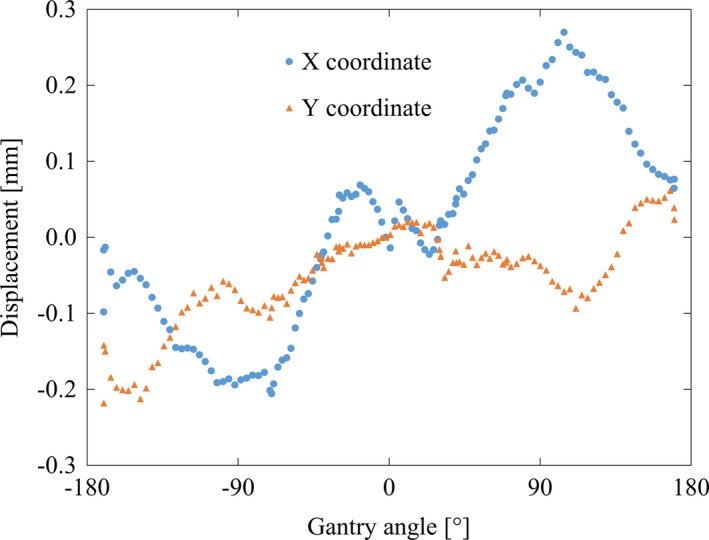
The outermost leaf pair excursion in the EPID with a clockwise gantry rotation in the range −180° to 180°. Circle and triangle show the averaged values for X and Y coordinate, respectively.

Figure 3 shows the calibration results for the misaligned EPID geometry. The COM of the X and Y coordinates was plotted according to each gantry angle. The outermost MLC pair formed of narrow slits moved a maximum 0.46‐mm resultant vector distance from the minimum and maximum positions on the EPID image during DWA irradiation. The calibration curve was acquired at multiple times. The standard deviation of calibration curve was less than 0.05 mm.

**Figure 4:**
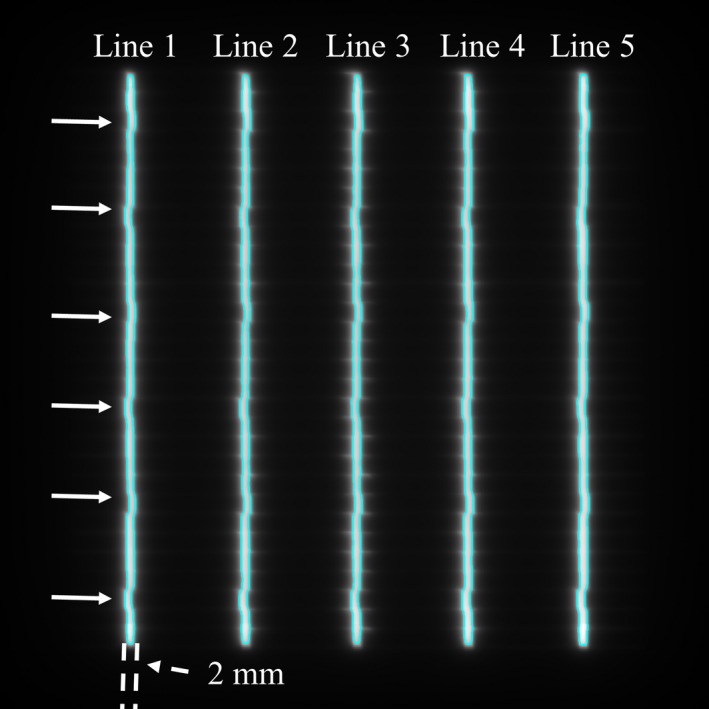
The integrated EPID images acquired during picket fence test at gantry angles of 0°. Intentional MLC positional errors of 0.5 mm were introduced. Arrows show MLC positions with intentional errors.

Intentional MLC positional errors within a range of 0.1–1.0 mm were detected in the EPID images. Positional errors of more than 0.5 mm were visually identified (Fig. 4). The analysis provided a result for the displacement of the MLC slit positions between those with and without an intentional error of 0.1 mm, which was difficult to confirm visually. The pixel size of the EPID was 0.18 mm, and the MLC slit profile between pixels was interpolated. Then, detected displacements were underestimated compared with given errors. However, it is notable that the intentional positional error of 0.1 mm, which is smaller than the EPID pixel size, could be identified. Using log data analysis, The MAE ± SD of MLC positional in static picket fence test were 0.00 ± 0.02 mm. Furthermore, 0.1 mm MLC positional errors with intentional error were detectable by using log data analysis.

#### 3.A.1 Test 1: Accuracy of the MLC positions

The profiles for DWA irradiation were in good agreement with those of the static gantry. Measuring the FWHM of the MLC slits in the EPID, the MAE ± SD of MLC slit widths was 0.1 ± 0.1 mm. It agreed within 0.20 mm of their exposed values. In addition, the MAE ± SD of displacement of the MLC between two slits at peak‐to‐peak was 0.2 ± 0.1 mm. It also agreed within 0.3 mm. Using log data analysis, the RMSE of the MLC, gantry, and ring positions was 0.03 mm, 0.10°, and 0.05°, respectively. The MAE ± SD of the MLC, gantry, and ring positions was 0.00 ± 0.02 mm, 0.05° ± 0.08°, and 0.00° ± 0.05°, respectively.

#### 3.A.2 Test 2: Accurate control of the dose rate, gantry speed, and ring speed

**Figure 5:**
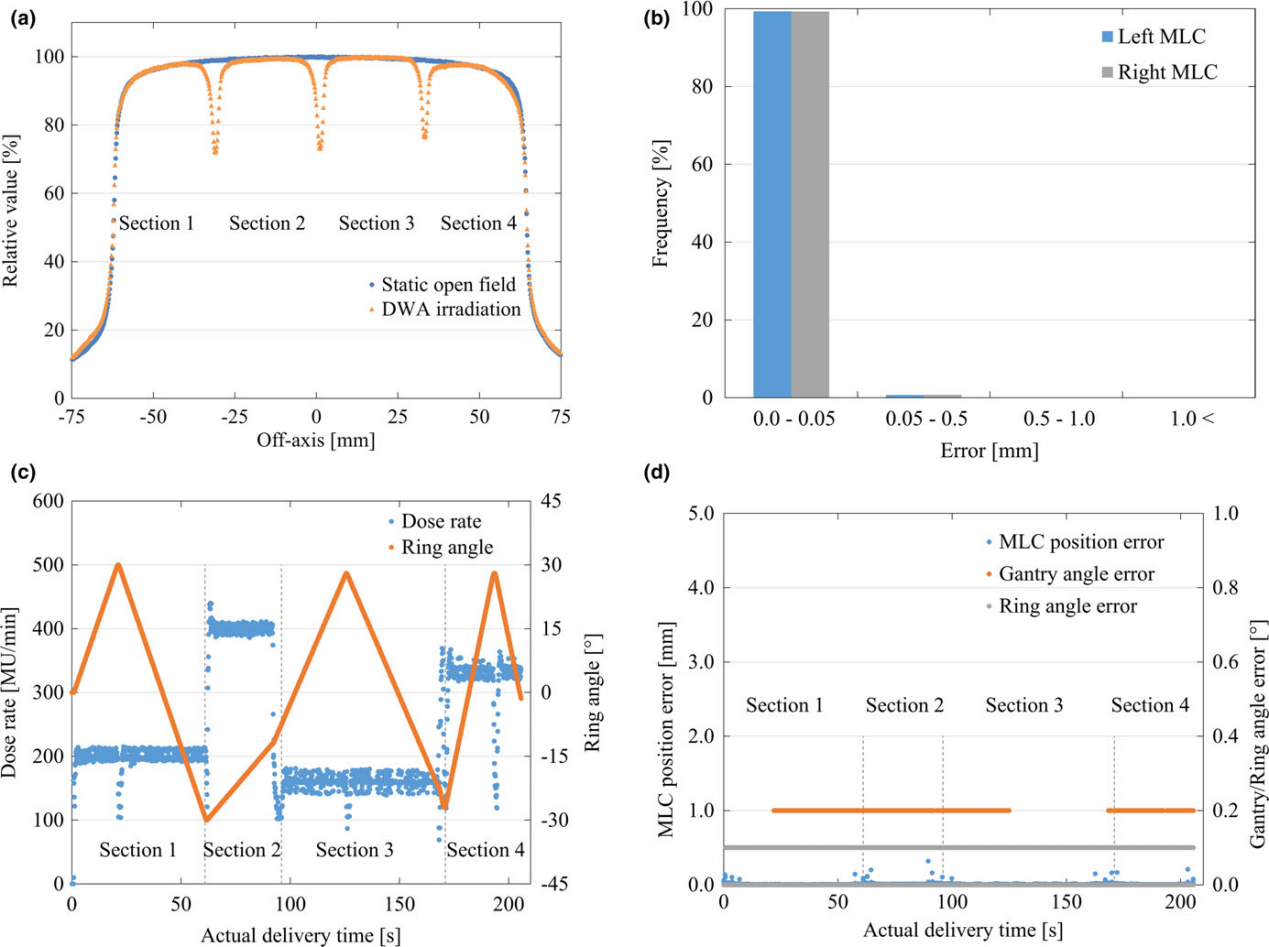
Comparison of the output constancy between the open field of the stationary gantry and DWA delivery with respect to Test 2. Illustration of (a) the output profile of different combinations of the dose rate and gantry and ring speeds, (b) mechanical MLC position errors using the log data, (c) the relationship between the actual dose rate and ring position based on the log data, and (d) the mechanical errors of the MLC, gantry, and ring positions using log data. Dotted lines show variable conditions for each section in Test 2. Abbreviation: DWA, Dynamic WaveArc; MLC, multileaf collimator.

With the exception of the gaps between the strips, the in‐field output profiles for the DWA and open field were closely matched. The averaged RMSE of the EPID profiles in the four strips was 0.4%, with values lying within the range −0.6% to 0.2% in the strips [Fig. 5(a)]. The MAE error ± SD of the EPID profiles in the four strips was 0.3% ± 0.3%. The RMSE of the log data was 0.02 mm, 0.14°, and 0.07° for the MLC, gantry, and ring positions, respectively. The MAE error ± SD of the log data was 0.01 ± 0.02 mm, 0.00° ± 0.14°, and 0.00° ± 0.07° for the MLC, gantry, and ring positions, respectively [Figs. 5(b)–5(d)].

#### 3.A.3 Test 3: Accurate control of the MLC leaf speed

**Figure 6:**
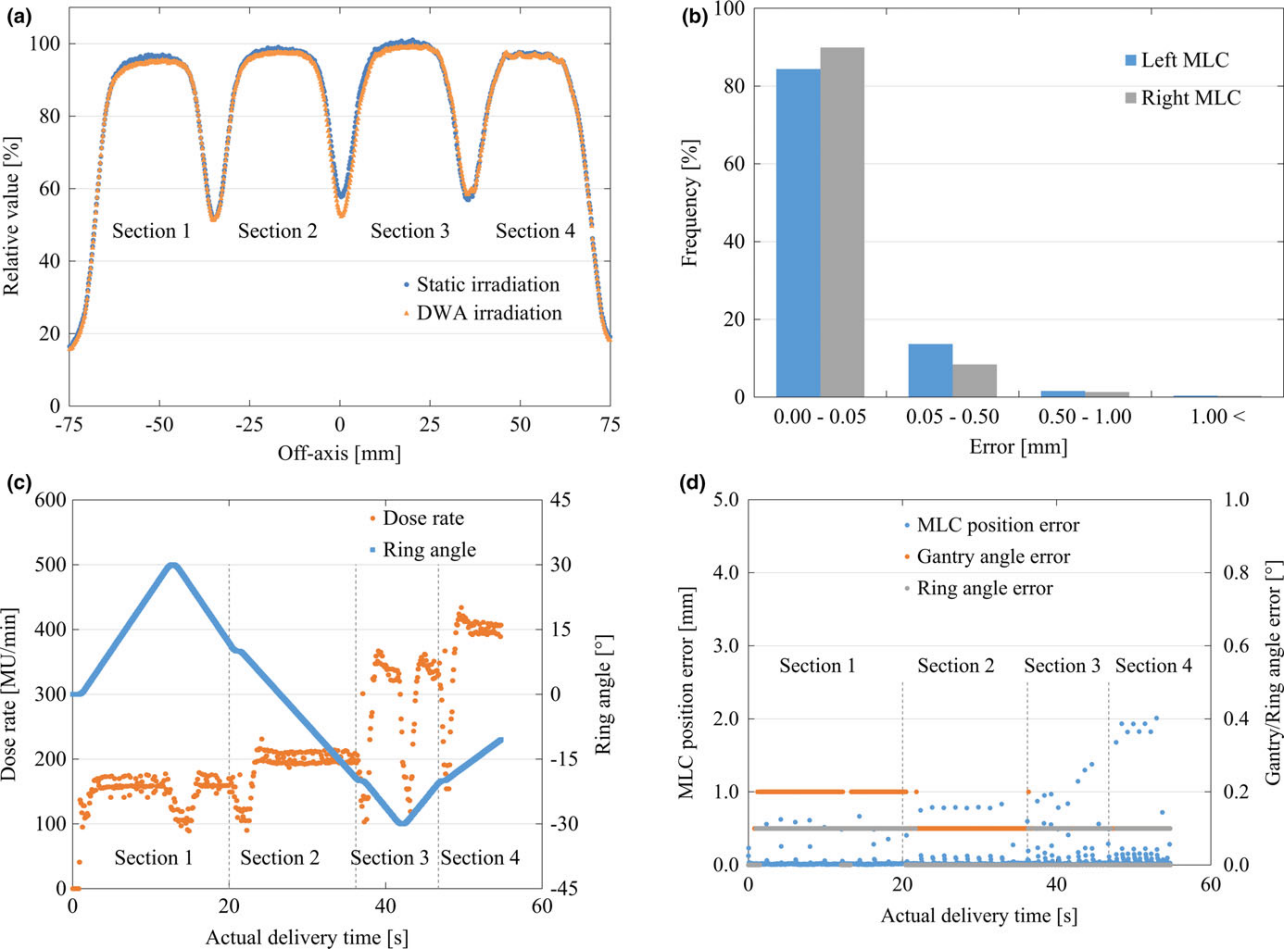
Comparison of output constancy in variable MLC speeds between the stationary gantry and DWA delivery with respect to Test 3. Illustration of (a) the output profile of different combinations of the dose rate, and gantry, ring, and MLC speeds, (b) mechanical MLC position errors using the log data, (c) the relationship between the actual dose rate and ring position based on the log data, and (d) the mechanical errors of the MLC, gantry, and ring positions using log data. Dotted lines show variable conditions for each section in Test 3. Abbreviation: DWA, Dynamic WaveArc; MLC, multileaf collimator.

The two measured profiles from the static gantry and the DWA modes were closely matched. The averaged RMSE of the four strips was 0.8%, with all deviations lying within the range −1.3% to 1.9% in the exposed field [Fig. 6(a)]. The MAE ± SD of the four strips was 0.7% ± 0.4%. The RMSE of the MLC, gantry, and ring positions in the log data was 0.10 mm, 0.12°, and 0.07°, respectively. The MAE error ± SD of the log data was 0.01 ± 0.19 mm, 0.01° ± 0.12°, and 0.00° ± 0.07° for the MLC, gantry, and ring positions, respectively [Figs. 6(b)–6(d)]. The greatest difference in the dose rate between the planned and actual values was observed at the moment the direction of ring rotation was reversed [Fig. 6(c)]. That is, dose rate modulation was slightly affected by the rotation of the ring incorporating mechanical stopping and a reversing motion. The MLC position error increased linearly with leaf speed. A maximum error of 2.01 mm was recorded when the direction of MLC movement of 4.0 cm/s was reversed [Fig. 6(d)].

## 4 DISCUSSION

The proposed method showed that the beam fluence detected by the EPID images was combined with log data used to assess output constancy and machine accuracy during DWA irradiation. It is important to check the performance of the machine for the accurate and precise delivery of radiation. American Association of Physicists in Medicine Task Group 142 (AAPM TG 142)^24^ and European Society for Radiation and Oncology (ESTRO) Booklet No. 9^25^ represented the tolerance on the accuracy of the MLC, gantry position, and output constancy. To evaluate these mechanical accuracies, combined analysis of the EPID and log data allows the easy verification of these items within a short period of time. Many studies on such evaluation methods using the EPID and log data have been reported,^26^, ^27^, ^28^, ^29^, ^30^, ^31^, ^32^ which indicated that EPID‐based machine QA for IMRT and VMAT was efficient. In this study, we devised a method to apply EPID‐ and log data‐based machine QA to advance noncoplanar irradiation such as the DWA technique. A test pattern suitable for DWA irradiation was developed, not for VMAT. In addition, the proposed method was simply performed and it is possible to comprehensively verify items given to irradiation accuracy of DWA. The results of this study showed the effectiveness of the developed QA procedure and its applicability to the clinical implementation of DWA irradiation.

The Vero4DRT incorporates the unique function of beam axis correction using the gimbal head. With this correction, the beam axis position relative to the center of the EPID images was displaced by up to 0.46 mm. To identify and correct the misalignment between the center of the EPID and beam axis, an automatic calibration method based on the outermost MLC slit aperture position was developed. Rowshanfarzad et al. took gantry sag into account for accurate pretreatment verification using the Winston–Lutz test method in EPID images.^33^ Zwan et al. presented a new detection method for collecting gantry sag using the EPID.^34^ However, these methods must incorporate preverification in order to validate the degree of sag. The advantages of our proposed calibration method are that the calibration is simple to perform and that it can be performed concurrently with the measurement of the proposed QA procedure. Moreover, outermost MLC was the static condition, and positional error of these leaf pairs were negligible small and did not affect the formed integrated image. In the log analysis, positional error of these leaf pairs was not detected. For the calibration of proposed method, additional independent checks of these outer leaf pairs is not particularly necessary. It is also available for VMAT verification with general treatment machines, in addition to DWA with the Vero4DRT.

Intentional errors were introduced into the MLC slit widths to assess the detection accuracy for the MLC position using EPID images. The results indicated that it was possible to detect errors smaller than the pixel size of the EPID. Ling et al. reported that intentional MLC positional errors larger than 0.5 mm were visible on film.^23^ Agnew et al. showed that EPID images (0.39 mm/pixel) detected 0.1‐mm intentional gap errors.^17^ Eckhause et al. examined whether or not a small MLC displacement (0.1–0.5 mm) could be detected by using EPID images (0.39 mm/pixel), and they were able to distinguish MLC deviations exceeding 0.3 mm, which is smaller than the EPID pixel size.^18^ Thus, subpixel estimation can be implemented using interpolated pixel values. In this study, detected displacements were underestimated compared with given errors. One of the reason for the underestimation of MLC slit width with intentional error less than pixel size is subpixel interpolation. The intentional errors were detected by FWHM in the slit. In order to calculate FWHM from the MLC slit profile, the values between pixels were obtained using linear interpolation. However, the EPID resolution of the Vero4DRT was 0.18 mm/pixel at the isocenter plane and this gave sufficient sensitivity to detect positional errors with submillimeter sizes. Furthermore, several researchers reported that Varian Dynalog files were not sufficient to detect MLC leaf position errors.^17^, ^35^ Zwan et al. showed that as the EPID measurements are a direct independent measurement of the MLC‐defined radiation field rather than log data.^19^ These results showed that log data must be carefully used to assess MLC position. In this study, we compared MLC slit widths in the integrated EPID image and ones in log data by using the static picket fence test without the intentional error. The result showed that the errors of MLC slit widths between planned and actual values were within 0.2 mm in EPID image and within 0.05 mm in log data analysis. Moreover, MLC slit width with 0.1 mm intentional error was detectable both analysis method. These results represented that the difference of MLC slit widths between EPID and log data was small and the reasonable agreement with EPID. Therefore, we confirmed that the detection accuracy of log data‐based analysis was less than 0.1 mm, which is same for EPID detection accuracy. Less than 0.1 mm detection accuracy of log analysis was sufficient in the clinical.

The picket fence test was successfully adapted for use in the QA of DWA irradiation in Test 1. By comparing irradiation profiles in the static gantry and DWA modes, the effect of simultaneous G/R rotation and leaf position accuracy was assessed. The difference in MLC width in the static gantry and DWA modes was less than 0.2 mm, which is less than 0.27 mm with the maximum MLC position deviation reported by Jørgensen et al.^26^ The RMSE in the MLC position based on log data was 0.03 mm, which is less than the 0.5‐mm deviation of the MLC position suggested by Ling et al.^23^ The displacement of the MLC between slits was <0.3 mm during DWA irradiation. The position which showed the largest error in the MLC width between slits coincided with the offset position. This is comparable to studies that showed that MLC position errors between slits were less than 0.5 mm at the offset position.^18^, ^23^ Hence, these results also indicate the effectiveness of the calibration method for the misaligned EPID geometry.

The output constancy was verified in Test 2 and Test 3. Test 2 varied the parameters of dose rate and G/R speed. The normalized output of the DWA was in good agreement with that of the open field with a static gantry, showing an RMSE of 0.4% in the exposed field. Machine uncertainty was also small in the log data analysis. Ling et al. showed that the mean deviation of the output constancy in a similar test was 0.7%^23^. ESTRO Booklet No. 9 proposed a confidence limit of ±3% in a variable dose rate and gantry speed test.^25^ Jørgensen et al. reported that the mean deviation for the dose rate versus gantry speed test was 2%.^26^ Compared to these reported results, our result indicated that the output constancy, including the effect of machine uncertainty, can be considered to be acceptable.

Likewise, the results of Test 3 showed that the RMSE for the output with variable MLC speeds was 0.8%. In comparison with a previous study,^23^, ^26^ our results for DWA verification showed sufficient accuracy. A decrease in the dose rate owing to ring inversion was found to have a slight impact on the output [Figs. 6(a) and 6(c)]. Even taking into account the effect of ring inversion, output variation was well controlled within 1%–2% in RMSE. According to Ling et al., the MLC leaf position error increases linearly with leaf speed^26^. Our result also showed that the MLC leaf position error was largest under the condition of maximum MLC speed (4.0 cm/s) according to the log data analysis [Fig. 6(d)]. Although MLC position errors were, on the whole, observed with reciprocating movement of the MLC; they were instantaneous errors and did not significantly affect the output [Figs. 6(a) and 6(d)]. In addition, the effect of the largest MLC errors at the maximum MLC speed on output was reduced because the maximum MLC speed would be rarely used during DWA irradiation. Thus, these errors were within the tolerance range supported by several reports.^23^, ^24^, ^25^

Utilizing the proposed method, it is possible to eliminate the time‐consuming work of the QA for the DWA technique. Irradiation of Tests 1, 2, and 3 was completed in less than 10 minutes. After that, automatic analysis was finished within at least five minutes by the use of in‐house software. This simple and efficient QA procedure is useful in the clinical application of DWA irradiation.

## 5 CONCLUSIONS

A simple and efficient QA method using the EPID and log data enables evaluation of the machine position and output under various DWA irradiation conditions. The proposed method is useful for the routine QA of DWA irradiation instrumentation.

## CONFLICT OF INTEREST

The authors have no relevant conflicts of interest to disclose.
